# Diagnosing Arterial Stiffness in Pregnancy and Its Implications in the Cardio-Renal-Metabolic Chain

**DOI:** 10.3390/diagnostics12092221

**Published:** 2022-09-14

**Authors:** Vladiana-Romina Turi, Constantin Tudor Luca, Dan Gaita, Stela Iurciuc, Izabella Petre, Mircea Iurciuc, Tunde Horvath, Dragos Cozma

**Affiliations:** 1 Department of Cardiology, “Victor Babes” University of Medicine and Pharmacy, 300041 Timisoara, Romania; 2 Research Center of the Institute of Cardiovascular Diseases Timisoara, “Victor Babes” University of Medicine and Pharmacy, 300041 Timisoara, Romania; 3 Department of Obstetrics and Gynaecology, “Victor Babes” University of Medicine and Pharmacy, 300041 Timisoara, Romania; 4 Department of Pharmacy, Faculty of Medicine and Pharmacy, University of Oradea, 410028 Oradea, Romania

**Keywords:** cardio-renal metabolic chain, pregnancy, arterial stiffness, pulse wave velocity

## Abstract

Cardio-renal and metabolic modifications during gestation are crucial determinants of foetal and maternal health in the short and long term. The cardio-renal metabolic syndrome is a vicious circle that starts in the presence of risk factors such as obesity, hypertension, diabetes, kidney disease and ageing, all predisposing to a status dominated by increased arterial stiffness and alteration of the vascular wall, which eventually damages the target organs, such as the heart and kidneys. The literature is scarce regarding cardio-renal metabolic syndrome in pregnancy cohorts. The present paper exposes the current state of the art and emphasises the most important findings of this entity, particularly in pregnant women. The early assessment of arterial function can lead to proper and individualised measures for women predisposed to hypertension, pre-eclampsia, eclampsia, and diabetes mellitus. This review focuses on available information regarding the assessment of arterial function during gestation, possible cut-off values, the possible predictive role for future events and modalities to reverse or control its dysfunction, a fact of crucial importance with excellent outcomes at meagre costs.

## 1. Introduction

Each link in the metabolic chain is equally important, and any disruption can lead to alterations in the pathophysiology, which can further develop into risk factors and pathological conditions. An altered metabolism is closely linked to chronic inflammation, oxidative stress, endothelial dysfunction, arterial stiffness and calcifications, hyperglycaemia and insulin resistance, increased body mass and visceral and subcutaneous fat, dyslipidaemia, subclinical atherosclerosis, and modified gene expression and signalling pathways at cellular and molecular levels to what is known as cardiovascular (CV) renal metabolic syndrome [1,2]. 

Cardio-renal metabolic syndrome (CMRS) is a group of interconnected and interactive maladaptive CV and kidney disease risk factors, including hypertension, insulin resistance, and dyslipidaemia, that are caused by overnutrition and obesity [3]. There are no specific diagnostic criteria. Classic cardio-renal syndrome (CRS) has been defined as an illness of the heart and kidneys whereby acute or chronic dysfunction in one organ may lead to acute or chronic dysfunction in the other [4]; it is divided into five types [5], revolving around the primum movens, the heart or kidney: type 1—acute cardiorenal syndrome, type 2—chronic cardiorenal syndrome, type 3—acute renocardiac syndrome, type 4—chronic renocardiac syndrome and type 5—with simultaneous cardiac and renal injury in various clinical syndromes (sepsis, infections, drug abuse, connective tissue disorders, etc.). In a meta-analysis and systematic review performed by Stoner et al., it was demonstrated that cardiometabolic risk factors were positively associated with the index for arterial stiffness carotid femoral PWV, blood pressure, impaired glucose metabolism, and metabolic syndrome [6].

Cardiometabolic disorders (CMDs), including coronary heart disease, stroke and diabetes mellitus, are the preponderant causes of morbi-mortality in women globally [7,8], and a possible reason for this is that women are under-diagnosed and undertreated and do not benefit from primary and secondary prevention like their male counterparts [9].

Certain risk factors (RF) such as diabetes mellitus and smoking have a more significant impact on women compared to men, and gender-specific aspects like complicated pregnancies and the female reproductive process increase the vulnerability to cardiovascular disease (CVD) [9]. 

The early identification and ongoing postpartum follow-up of women with pregnancy complications such as hypertensive disorders of pregnancy (HDPs) and gestational diabetes mellitus (GDM) may offer opportunities for prevention or help delay the onset of CMDs [10]. Maternal morbidity and mortality rates have been escalated due to the advanced age at which pregnancy occurs and pre-existing pathological conditions [11]. 

Several risk factors and diseases are more encountered with increasing age, thus elevating the risk for complications during gestation. Older maternal age is linked to higher body mass [12], dyslipidaemia [13], glucose intolerance [14], decreased physical activity [15], modified vasculature walls [16], high blood pressure [17] and kidney disease [18], a constellation known as cardio-renal metabolic syndrome. 

The threshold for advanced maternal age is considered 35 y and over [19]. Pregnancy at an elderly age is related to poor maternal and perinatal results [20]. It was demonstrated that women over 35 have a significantly higher mortality risk than those under 35, increasing exponentially with the age gap, twice for the 35–39 age group, 4 times higher for those between 40 and 44 and 11 times higher for those 45–54 years [11]. 

Gestation could trigger or worsen a chronic illness and enhance mental and physical stress, thus increasing the maternal needs with a detrimental effect on the perinatal results, and this is why it is mandatory to explore and treat early [21]. 

One of the most important causes of morbi-mortality during pregnancy is represented by pre-eclampsia, occurring in 3–10% of all cases worldwide [22], and this is responsible for maternal deaths every minute. There is a constant increase in the cases of women with chronic conditions such as diabetes, high blood pressure and other CVDs who opt for pregnancy, with a more than four-fold increase in the past two decades [22]. Moreover, usually, these conditions overlap, having a cumulative effect. 

Regardless of the age gap, pregnant patients are complex, and many things are yet to be answered regarding particular mechanisms and pathophysiology. The entire maternal body suffers modifications during gestation [23,24,25]. The maternal physiological alterations prepare the body for the pregnancy period and birth process. Insufficient or inappropriate adaptations can lead to CV, metabolic and renal consequences in the short and long term for both mother and foetus [26]. 

There is compelling evidence that maternal vascular deficiencies have a role in pregnancy-related illnesses such as pre-eclampsia and intrauterine growth restriction [27]. Vascular alterations can be strictly pregnancy-induced, or they can be a consequence of pre-existing conditions, and other systems also suffer alterations during the gestation period for the proper development of the foetus. 

Peripheral vasodilatation is secondary to haemodynamic overload [28], and arterial function and stiffness changes throughout the gestational period [29]. Arterial stiffness refers to the rigidity of the arterial wall [30], and it offers an insight into vascular function. Arterial stiffness is an age-related phenomenon, and it is a secondary effect of multiple diseases and risk factors such as hypertension, dyslipidaemia [31], hyperglycaemia/diabetes [32] and inflammation [33]. Studies have shown that women with pre-eclampsia have increased arterial stiffness compared to normotensive patients, and pulse wave velocity can be utilised as a predictor for developing pre-eclampsia [34]. 

Pre-eclampsia is an underdiagnosed, under-recognised risk factor for developing subsequent CV, metabolic and renal disease in women; it is a conjunction between pre-existing vascular RF and endothelial dysfunction due to placental mediated anti-angiogenic factors, and it is considered that pre-eclampsia is a type 5 cardio-renal syndrome [35], which often coexists with diabetes. 

Increased arterial stiffness precedes high blood pressure onset, and it is directly related to the degree of hypertension and a valuable predictor of CV morbidity and mortality [36]. It is a reliable indicator of morbidity and mortality and has become a regulation of the international guidelines, where it is recommended to assess arterial stiffness through pulse wave velocity (PWV) and pulse pressure (PP) as ways of detecting asymptomatic hypertension-mediated organ damage (HMOD) [37]. The gold standard of arterial stiffness is carotid-femoral pulse wave velocity, and it has been proven that arterial stiffness assessed through pulse wave velocity could predict pregnancy outcome [38]. 

Although PWV is recommended for the general population, there is no precise specification in this matter for the pregnancy population, and no standard cut-off values [29]. 

In the present paper, the main aspects regarding the early detection and management of gestational women with a high risk for cardiometabolic disease are presented, focusing on the assessment of arterial stiffness as a shared feature of all risk factors and pathologies involved. This review also focuses on arterial stiffness and its impact on the mother’s body, the evolution of the foetus, and the capacity to predict a future cardio-renal metabolic syndrome. 

## 2. Physiological Modifications during Pregnancy

**Figure 1:**
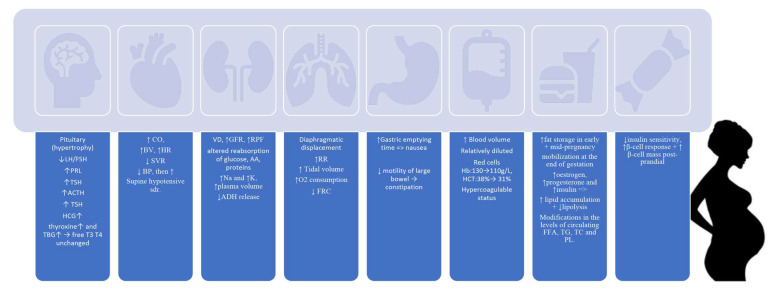
Physiological modifications occur during pregnancy in the endocrinological, cardiovascular, renal, respiratory and gastro-intestinal systems, with adaptations of the haematological area and lipid and glucose metabolism. LH = luteinising hormone, FSH = follicle-stimulating hormone, PRL = prolactin, ACTH = adrenocorticotropic hormone, TSH = thyroid-stimulating hormone, TBG = thyroid-binding globulin, CO = cardiac output, BV = blood volume, HR = heart rate, SVR = Systemic Vascular Resistance, BP = blood pressure, VD = vasodilatation, GFR = glomerular filtration rate, RPF = renal plasmatic flow, AA = amino acids, Na = sodium, K = potassium, RR = respiratory rate, FRC= functional residual capacity, Hb = haemoglobin, HCT = haematocrits, FFA = free fatty acids, TG = triglycerides, TC = total cholesterol, PL = phospholipids.

Modifications during pregnancy are diverse [39], ranging from anatomical to functional and behavioural, with the most significant changes being in the hormonal, CV, renal and respiratory systems, the haematological area and the lipid and glucose metabolisms (Figure 1). There is pituitary hypertrophy, with decreased LH/FSH and increased PRL, TSH, ACTH, HCG, thyroxine and TBG, whereas levels of free T3 and T4 remain unmodified [40]. 

The CV system enhances the heart rate, blood volume and cardiac output, and causes a marked decrease in systemic vascular resistance [41]. Blood pressure initially shows a reduction, and afterwards, it increases progressively [42,43]. Vasodilation begins from week 5 [44] and continues throughout the pregnancy due to increased oestrogen biosynthesis [45], resulting in a a 20–50-fold enhancement in the uterine blood flow towards the end of gestation. Recent evidence has proven that vasodilation could be secondary to the proangiogenic vasodilatory effect of hydrogen sulphide (H_2_S) [46] synthesised through selective upregulation from L-cysteine by cystathionine-β. Loerup et al. performed a systematic review and meta-analysis in 2019 based on 39 studies following the blood pressure trends during gestation [47], and observed that the lowest mean SBP was at 10 weeks, DBP at 21 weeks and HR increased progressively, and also that the DBP was higher when measured by manual devices versus classic. Surprising data have come from Vivek et al., who emphasised in their study that BP assessed under controlled conditions showed a progressive rise from baseline, without the well-known second-trimester decrease [48] seen in the majority of the existing data in the literature.

In the renal system, marked vasodilatation enhanced the glomerular filtration rate and renal plasmatic flow, and decreased the reabsorption of amino acids, protein and glucose [49]. The sodium and potassium levels are augmented, while the antidiuretic hormone decreases its activity and the plasma volume increases [50]. The respiratory system suffers modifications through diaphragmatic displacement, increased respiratory rate, tidal volume and O_2_ consumption, whereas the functional residual capacity decreases [51]. The gastro-intestinal system works slower, with prolonged gastric emptying, nausea, decreased motility and secondary constipation [52]. Haematological changes include enhanced blood volume, diluted and reduced haemoglobin values, haematocrit reduction, and a hypercoagulable status [53]. Lipid metabolism changes consist of an augmentation of the fat storage at the beginning and mid-pregnancy intervals, with their mobilisation towards the end of gestation [54]. The enhanced levels of oestrogen, progesterone and insulin have led to increased lipid accumulation and decreased lipolysis [54,55].

Moreover, the circulating levels of total cholesterol, triglycerides, phospholipids and free fatty acids are elevated [56]. Glucose metabolism alterations include decreased insulin sensitivity and an increase in β-cell response and β-cell mass post-prandially [57,58]. All the above are mandatory changes for the proper development of the foetus and favourable outcomes for both mother and offspring, and any disruption can lead to severe consequences for both the mother and the foetus. Any disruption in any of the links in the cardiovascular renal metabolic chain can lead to severe consequences in both the mother and foetus.

## 3. Arterial Stiffness in General Population

Apart from the irreversible and unstoppable process of aging, there are other risk factors, considered “classic”, such as hypertension, dyslipidaemia, diabetes, obesity, etc., that also contribute to early vascular changes, starting from the smallest up to the largest vessels [59]. The modifications are both structural and functional, and the responsible factors include oxidative stress, mitochondrial dysfunction, altered resistance to molecular stressors, chronic low-grade inflammation, genomic instability, cellular senescence, epigenetic alterations, loss of protein homeostasis, deregulated nutrient sensing, and stem cell dysfunction in the vascular system [60], with an increase in collagen fibres and a decrease in elastin, and the artery becomes stiffer, opening the door for atherosclerosis. 

There are various invasive and non-invasive ways to assess arterial function, and the most utilised and preferable modalities include carotid-femoral pulse wave velocity (cf-PWV), brachial-ankle PWV (ba-PWV), the Cardio Ankle Vascular Index (CAVI), pulse wave analysis (PWV) and augmentation index (Aix) [61]. All parameters rely on the velocity of the pulse wave throughout the arterial tree, but the gold standard recommended in the guidelines is the cf-PWV [62]. CfPWV predicts CV events and all-cause mortality in patients with hypertension, chronic kidney disease and elderly age, and also in the general population [63]. The 2018 Practice Guidelines for the management of arterial hypertension of the European Society of Hypertension and the European Society of Cardiology recommend assessing the CV risk using the SCORE diagram (unless the patient is already at high or very high risk) and identifying hypertension-mediated organ damage (HMOD) through 12-lead ECG, urine albumin:creatinine ratio, blood creatinine and GFR, fundoscopy, and more meticulously through echocardiography, carotid ultrasound, PWV, abdominal ultrasound, ankle-brachial index, cognitive function tests and brain imaging [64]. 

PWV can be positively influenced by controlling BP values and through medical treatment (beta-blockers, angiotensin converting enzyme inhibitors and angiotensin receptor blockers, statins) [59]. Additionally, PWV can be negatively affected by several pathologies involving chronic inflammation (HIV, rheumatoid arthritis, vasculitis) and by their treatment also [65]. 

The clinical importance of assessing the target organ damage through measuring PWV consists in the capacity to predict future events, thus offering the chance to prevent complications. Nonetheless, further studies are necessary to prove its modifications throughout life and the impact of medical treatment on BP versus PWV, as well as the consequences in the long term. 

## 4. Arterial Stiffness in Normal Pregnancies

In order to understand the modifications of the arterial walls during the gestation period, it is crucial to know the general physiology of the arteries. 

The main vessel, the aorta, divides itself into a so-called “arterial tree” with larger and smaller branches ending with capillaries [66]. Because cardiac ejection has a variable, pulsatile character, blood pressure and blood flow vary significantly through the entire body [67]. The high elastin content of the aorta makes it paramount in buffering blood pressure variations [68]. The smaller arteries and arterioles play another role in adjusting the smooth muscle’s tone [69]. Arterial wave reflection, endothelial function, and arterial stiffness are indicators of vascular tone modulation and large-artery elasticity, and thus, arterial function [70]. Arterial stiffness represents the rigidity of the arterial wall and is a measure of the vascular function [71]. 

Arterial stiffness can be assessed through various modalities and techniques. The gold standard of arterial stiffness measurements is represented by the aortic pulse wave velocity (PWV), and for the PWV assessment, velocity is expressed as a change in distance over the change in time (V = ΔD/ΔT), whereas the distance is measured at two sites along a single vessel or two separate sites in the arterial tree [72]. The measurement of PWV is a simple and reproducible technique used to assess vascular stiffness [73]. There are several non-invasive modalities through which PWV can be measured, such as tonometry, oscillometry, ultrasound, MRI, or via piezo-electronic pressure transducers, and each has its advantages, challenges, and disadvantages [74]. The most widely utilised method for estimating PWV is the foot-to-foot method: the time difference between the feet of the two pulse waveforms [75]. The reference values for PWV come from multicentre registries from different countries and continents [76]. Additionally, arterial stiffness is the earliest proof of arterial dysfunction and is a well-known independent predictor of CV morbidity and mortality [77]. The most studied classic RFs influencing arterial stiffness are age, hypertension, dyslipidaemia, diabetes, and smoking [78,79,80]. 

During pregnancy, there is a significant change in arterial stiffness compared to pre-pregnancy [81]. Physiological gestation is associated with modified arterial stiffness, central aortic pressure and wave reflections, and increased flow-mediated dilation [82]. Arterial stiffness and undulant evolution vary throughout pregnancy, according to the trimester [29]. Mahendru et al. proved a decrease in BP, Aix, and peripheral vascular resistance (PVR) in early pregnancy, with a peak in the second trimester, and PWV decreases from the second half of the gestation period. They also noticed that BP and Aix were lower after birth than at the pre-gestation age [83]. 

A 10-year study compared the arterial function in healthy pregnant women versus non-pregnant women, and noticed a decrease in both Aix brachial and PWV in the second and third trimesters, followed by a postpartum increase [29]. The published data have been used to evaluate the association between foetal growth and arterial stiffness in 50 pregnant women with normal blood pressure, and observed a direct and significant correlation between PWV, birth weight centile and growth after delivery independently of mean arterial pressure, emphasising that arterial stiffness represents a reflection of the CV maternal system’s adaptation more accurately than blood pressure [84]. Increased arterial stiffness is associated with multiple maternal and foetal pathologies in the short/ long term, e.g., young adults born pre-term have alterations in arterial distensibility [85]. The main question regarding arterial stiffness during normal pregnancy is related to the cut-off values. 

## 5. Complicated Pregnancies and the Interconnected Consequences

### 5.1. Hypertensive Disorders during Pregnancy 

**Figure 2:**
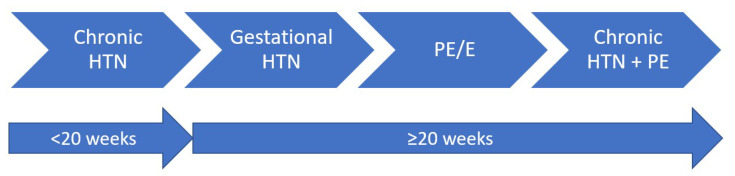
The classification of pregnancy-related hypertensive disorders; HTN = hypertension; PE = pre-eclampsia; E = eclampsia.

Hypertension during gestation is the most commonly encountered disorder, affecting approximately one-tenth of pregnancies [86], with possibly fatal consequences for both mother and foetus. It is responsible for >16% of maternal deaths [87], and survivor women are predisposed to premature CVD. Maternal mortality has increased by 26.6% in the USA, from 18.8 per 100,000 live births in 2000, to 23.8 per 100,000 live births in 2014 [88]. Pregnancy-related hypertensive disorders (Figure 2) include chronic hypertension, gestational hypertension, pre-eclampsia/eclampsia and chronic hypertension with superimposed pre-eclampsia [89]. 

Hypertension is defined as an SBP ≥ 140 mmHg or DBP ≥ 90 mmHg, and its values should be confirmed over four hours with repeated measurements or after overnight rest to determine if there is veritable hypertension [90]. Severe hypertension is classified as an SBP ≥ 160 mmHg, or DBP ≥ 110 mmHg or above [90], and requires urgent management in hospital settings. Chronic hypertension is associated with adverse maternal and foetal outcomes, and can be managed by exerting strict control over the blood pressure values (110–140/85 mmHg), closely assessing foetal growth and the possible complications in the outpatient setting [91]. 

Women with chronic hypertension could be on antihypertensive treatment before pregnancy, and even conceive while under treatment, but their monitoring plan needs to include the assessment of complete blood count, urea, creatinine, electrolytes, liver functions tests, uric acid, urinalysis and microscopy, urine protein–creatinine ratio, and ECG [92]. We must remember that some drugs, such as ACE inhibitors or angiotensin receptor blockers, are contraindicated during pregnancy. In contrast, others are not recommended and should be avoided, such as diuretics, beta-blockers (other than labetalol) and calcium-channel antagonists (other than nifedipine and diltiazem) [93]. Nonetheless, some drugs can and should be used for controlling BP values during gestation, such as labetalol, nifedipine, methyldopa, hydralazine and prazosin, with caution and attention to side effects [93]. First-line options for severe hypertension usually include intravenous (IV) labetalol, oral nifedipine or IV hydralazine, and the avoidance of sublingual nifedipine [94].

Gestational hypertension occurs after 20 weeks without proteinuria or biochemical or haematological alterations, and a quarter of patients (especially those who present at <34 weeks) will develop pre-eclampsia and have substandard results, with >500,000 foetal and neonatal deaths, and >70,000 maternal deaths [92]. Proteinuria is not a sine qua non condition for the diagnosis of pre-eclampsia, as the diagnosis implies the existence of newly occurring HTN after 20 weeks of gestations in the presence of proteinuria and/or proof of acute maternal kidney injury (AKI), liver dysfunction, neurological traits and haematological features (thrombocytopenia or haemolysis), or foetal growth restriction [95]. 

Signs and symptoms of pre-eclampsia include headache, visual disturbances, epigastric or right upper quadrant discomfort and oedema [92]. Some types of pre-eclampsia can trigger during birth or in the early postpartum period. The HELLP syndrome (haemolysis, elevated liver enzymes, low platelet number) is a severe morbidity with high mortality, and a complication of pre-eclampsia [95]. The enhanced prevalence of hypertensive disorders during pregnancy could result from multiple factors, such as advanced age, the use of assisted reproductive technology [96] and unhealthy lifestyle [97]. 

### 5.2. Obesity

Data from the National Health and Nutrition Examination Surveys (NHANES) sustain that obesity (BMI > 30) has increased from 14.5% (between 1976 and 1980) to 36.5% (between 2011 and 2014) in the US, and 70% of US adults aged ≥20 years were either overweight (defined as BMI >25) or obese (BMI > 30) between the years of 2013 and 2014. Overall, the prevalence of obesity is higher in women (38%) than in men (34%) [98]. The World Health Organization considers this trend as a “global epidemic” and a severe threat to public health.

In the most straightforward approach, obesity is a BMI greater than or equal to 30 kg/m^2^, and extreme obesity is a BMI greater or equal to 40 kg/m^2^ [99]. In a more detailed classification according to WHO and NICE [100,101] (Table 1), there are several categories with increasing complications directly related to the value of the BMI. However, BMI has its limitations because not just the numbers are essential. However, the distribution of the adipose tissue is more significant because abdominal obesity is linked to increased insulin resistance and morbi-mortality compared to adiposity on the thighs and hips, a fact that cannot be assessed through BMI alone. Therefore, waist circumference was added as a more accurate assessment of visceral fat and its risks [102]. 

According to the Obesity Medicine Association, obesity is defined as “a chronic, progressive, relapsing, and treatable multi-factorial, neurobehavioral disease, wherein an increase in body fat promotes adipose tissue dysfunction and abnormal fat mass physical forces, resulting in adverse metabolic, biomechanical, and psychosocial health consequences” [103]. The prevalence of maternal increased body mass index is increasing worldwide, especially in developed and developing countries, with detrimental consequences on both mother and foetus [104]. Maternal obesity is now considered one of the most commonly encountered risk factors seen in obstetric practice [105]. 

Obesity is associated with fertility issues, prolonged time to successful conceiving, and the following comorbidities—type 2 diabetes, chronic high blood pressure, early pregnancy loss, congenital foetal malformations, large for gestational age babies, shoulder dystocia, premature birth and stillbirth [106]. Towards the end of the gestation period, complications such as pre-eclampsia and gestational diabetes could occur, with consequences even after birth [105], and the delivery process could become problematic due to a haemorrhage. Obesity is a known risk factor for various pathologies such as gestational hypertension, diabetes, pre-eclampsia, caesarean-delivery and hydramnios [105]. Apparently, in overweight patients, pre-eclampsia could result from endothelial dysfunction and systemic inflammation due to insulin resistance caused by adiposity [107]. 

Bodnar et al. discovered that almost 1/3 of the total effect of the body mass index on pre-eclampsia is mediated through inflammation and triglycerides levels [108]. Numerous studies have explored the relationship between body mass index and pre-eclampsia onset [109]. Getahun and collaborators found that obesity/overweight in both pregnancies was correlated with the enhanced risk of pre-eclampsia in the following pregnancy, and that in African American race, decreasing BMI to a normal status was not followed by a decrease in the risk of pre-eclampsia [109]. 

Sattar et al. proved that waist circumference up to 16 weeks gestation can predict pregnancy-induced hypertension and pre-eclampsia. Therefore, waist circumference could form the basis for health promotion, involving raising awareness of the importance of or urging weight reduction for women planning pregnancies [102]. Several studies have demonstrated significant maternal health benefits for obese pregnant women who gain less than 5 kg, or even lose weight, during gestation [110,111]. However, although gaining less than 5 kg during gestation has clear advantages for peripartum disorders, it was associated with an increase in multiple risks for the neonate [112].

### 5.3. Dyslipidaemia

There are physiological increases in total cholesterol (TC) and triglycerides (TG) during pregnancy [113]. Some authors define dyslipidaemia (DLP) in pregnant women when TC, LDL and TG concentrations are above the 95th percentile (p95%), and HDL concentration is below the 5th percentile (P5%) for gestational age (GA) [114].

Dyslipidaemia has been linked to several complications for mother and child during and after pregnancy [115]. Gestational dyslipidaemia is linked to metabolic comorbidities such as gestational diabetes [116] and obesity [117], and is a risk factor for developing acute pancreatitis [118], pre-eclampsia [119] and pre-term birth [120]. 

Mahdenru et al. observed that lipid components (HDL-c and LDL-c) decreased at the beginning of the process, and the serum creatinine level decreased [83]. Dyslipidaemia is closely related to CV and renal impairment, and is linked to increased arterial stiffness (AS), which may lead to hypertension [121]. 

Another factor that could influence the lipid profile is second-hand smoking, which can significantly decrease HDL cholesterol [122]. In observational and more extensive population cohort studies in average women before pregnancy, increasing maternal triglycerides in early pregnancy is associated with increased rates of pre-eclampsia and GDM, and enhanced triglyceride levels in early pregnancy could increase the risk of gestational diabetes mellitus and pre-eclampsia [123]. 

The consequences do not occur only during the pregnancy and the immediate postpartum period, but also in the long run: for the mother the risk includes the development of dyslipidaemia in postpartum decades, and for the foetus, there is a risk of being born large for gestation age and developing atherosclerosis later in adult life [124]. Even though the gestational status is a risk factor for dyslipidaemia, the lipid profile is not included in the national routine obstetrical examination [125]. There is a lack of consensus regarding the cut-off values for the lipid profile according to the weeks/trimesters of pregnancy, and there are no standardised diagnostic criteria and no screening programs [126]. 

### 5.4. Diabetes

Gestational diabetes mellitus (GDM) is a pregnancy-related pathology defined as “diabetes that is first diagnosed in the second or third trimester of pregnancy that is not overt diabetes” (using a newer definition following the guidelines of the American Diabetes Association, 2021) [127]. Gestational diabetes mellitus (GDM) is the most frequent disease among pregnant women, affecting 15–25% of pregnancies worldwide [128]. According to the newest International Diabetes Federation (IDF) data, gestational diabetes mellitus occurs in approximately 14% of pregnancies worldwide, representing approximately 18 million births annually [129].

The consequences of GDM are both short- and long-term. GDM is associated with increased risks of adverse perinatal outcomes, and a study performed in 2008 showed that hyperglycaemia in pregnancy, less severe than overt diabetes, is independently associated with an increased risk of adverse maternal and neonatal outcomes [130]. 

Additionally, the latest research emphasizes that gestational diabetes is also independently a risk factor for cardiometabolic diseases of the mother and offspring [128]. 

### 5.5. Kidney disease

**Figure 3:**
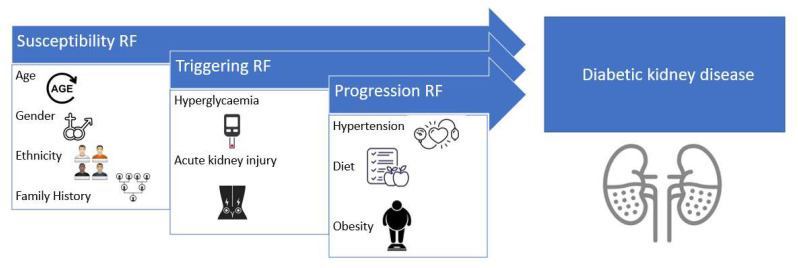
Risk factors for developing diabetic kidney disease.

Chronic kidney disease (CKD) is estimated to affect 3% of pregnant women, and it will increase further in the future due to advanced maternal age and obesity [131]. Pregnancy can accelerate the decline in kidney function in patients with chronic kidney disease [132], CVD being the leading cause of mortality for patients with kidney disease [133]. A possible explanation for this high rate of cardiovascular mortality in this category of patients is represented by the presence of co-occurring traditional risk factors (Figure 3) such as high blood pressure, diabetes mellitus, dyslipidaemia, and a unique feature of these patients—altered bone mineral metabolism and secondary vascular calcification [134]. In patients with CKD, there is an increased risk of CVD with manifestations such as coronary artery disease [135], heart failure and sudden cardiac death [136]. 

Moreover, hypertensive disorders of pregnancy (HDP) (pre-eclampsia, gestational hypertension) are associated with an increased risk of end-stage kidney disease (ESKD) [137], particularly hypertensive or diabetic forms of CKD, and the risk is enhanced after pre-term pre-eclampsia, recurrent pre-eclampsia, or pre-eclampsia complicated by pre-pregnancy obesity. 

## 6. Pre-Eclampsia—A Type 5 of Cardio-Renal Syndrome

Pre-eclampsia is a multisystem vascular disorder, and a leading cause of illness and mortality in pregnant women and their offspring. Its prevalence in Europe ranges from 2.8% to 5.2% [138]. The International Federation of Gynaecology and Obstetrics (FIGO) released 2019 data in which they revealed that on a global scale, 76,000 women and 500000 babies die each year from pre-eclampsia [139]. PE is responsible for 50,000–60,000 deaths annually worldwide [140].

According to the American College of Obstetricians and Gynaecologists (ACOG) and the International Society for the Study of Hypertension in Pregnancy (ISSHP), pre-eclampsia is a pregnancy-related disorder consisting of new-onset hypertension, usually after 20 weeks of gestation and proteinuria, or other end-organ damage occurring [141]. 

PE can be divided according to its delivery into four categories: early-onset PE (delivery at <34^+0^ weeks of gestation), pre-term PE (delivery at <37^+0^ weeks of gestation), late-onset (delivery at ≥34^+0^ weeks of gestation) and term PE (delivery at ≥37^+0^ weeks of gestation) [139]. Pre-eclampsia’s pathophysiology is complex, with implications in placental, vascular, renal, and immunological domains. However, its aetiology, pathogenesis and pathophysiology are poorly understood. It has been stated that it is a two-stage disease with a disbalance between angiogenic and anti-antigenic factors [142]. 

**Figure 4:**
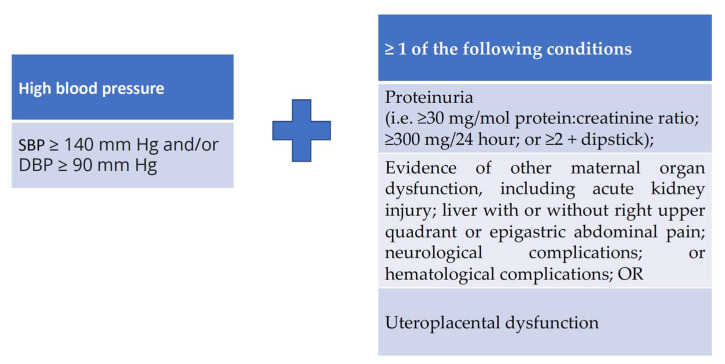
Diagnosis criteria for pre-eclampsia. SBP = systolic blood pressure, DBP = diastolic blood pressure.

The definition of PE (Figure 4) implies the existence of high blood pressure and at least one of the following conditions: proteinuria, maternal organ dysfunction (acute kidney injury, liver injury, neurological complications, haematological complications) or uteroplacental dysfunction [139]. There is a close relationship between pre-eclampsia and future CV and kidney disease [35]. It is generally acknowledged that there are two different types of pre-eclampsia: an early-onset or placental type and a late-onset or maternal type [143], the first one characterised by a low-output/high-resistance circulation and the other type as a high-volume/low-resistance circulation, with worse outcomes for the first one. 

With both types, there are several alterations in the heart, arteries, veins, and kidney functionality. There is a gestation-induced worsening of subclinical pre-existing chronic CV dysfunction in early-onset pre-eclampsia, thus sharing the pathophysiology of cardiorenal syndrome type II, and with acute volume overload decompensation of the maternal circulation in late-onset pre-eclampsia, thus sharing the pathophysiology of cardiorenal syndrome type I. In contrast, cardiorenal syndrome type V is consistent with the process of pre-eclampsia superimposed upon clinical CV and/or renal disease, alone or as part of a systemic disorder [143].

The risk factors for developing PE (Table 2) can be divided into minor and major. The minor risk factors include advanced age of the mother, nulliparity, family history of PE, short and long intervals between pregnancies, using assisted reproductive technologies, obesity, and ethnicity. Major risk factors include pre-existing chronic hypertension, renal disease, personal history of PE, and autoimmune disease [144,145]. Additionally, eclampsia is represented by the development of grand seizures in women with pre-eclampsia [143]. 

## 7. Arterial Stiffness in Complicated Pregnancies

**Figure 5:**
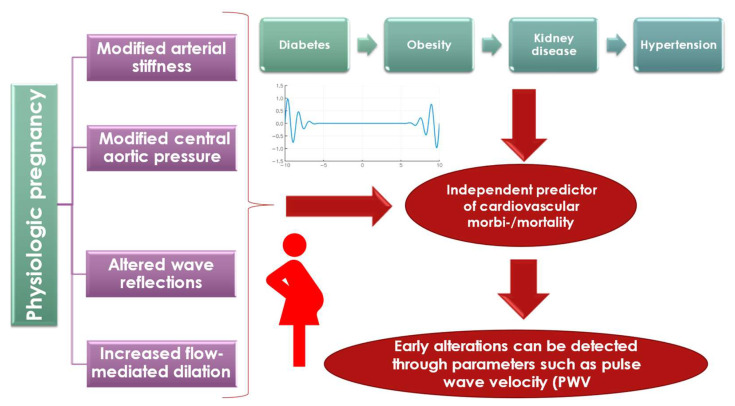
The interconnections between physiological vascular modifications, arterial stiffness and risk factors/pathologies (diabetes, obesity, kidney disease, hypertension).

As previously mentioned, physiological modifications during gestation, increased arterial stiffness, and risk factors and pathologies are interconnected (Figure 5). In normal pregnancies, there is a modification of the arterial stiffness parameters compared to the non-pregnant state. Nonetheless, these modifications are steeper in complicated pregnancies such as diabetes, obesity, kidney disease or hypertension, and they occur before the first signs of clinical manifestations; as already proven, PWV is an independent predictor of cardiovascular morbi-mortality. 

Arterial stiffness can be considered a precursor of cardiovascular disease [146,147] and pregnancy complications. Wen-Feng Li et al. conducted a systematic review and meta-analysis on the association between central haemodynamics and the risk of all-cause mortality and cardiovascular disease. They observed that the adjusted pooled hazard ratio of total cardiovascular events was 1.10 for a 10 mmHg increase in the central systolic pressure, 1.12 for a 10 mmHg increase in the central pulse pressure and 1.18 for a 10% increase in the central augmentation index, thus proving that central hemodynamic variables are independent predictors of cardiovascular disease and all-cause mortality, and thus offering an excellent opportunity for better clinical practice [148]. Furthermore, Hausvater et al. conducted a systematic review and a meta-analysis in which they included twenty-three relevant studies, and they observed an essential enhancement in all arterial stiffness indices combined in patients with pre-eclampsia vs. normal pregnancies, with different degrees in pregnant hypertensive women vs. pre-eclamptic women. Moreover, they finally concluded that arterial stiffness measurements could help predict pre-eclampsia, and may play a role in the increased risk of future CV complications in women with a history of PE [149]. Before gestational hypertension sets in, there is a prior stage consisting of modifications in the arterial wall leading to increased arterial stiffness [150]. These early alterations can be detected through arterial stiffness parameters’ measurements, such as pulse wave velocity (PWV) and augmentation index (Aix), and afterwards, proper intervention can prevent further process advancement [151]. A few articles have approached the close link between arterial stiffness and hypertension in pregnant patients with pre-eclampsia and eclampsia [67,152]. It was found that marked changes [153], such as the lack of mid-trimester BP lowering, could signify an early sign of arterial stiffness and pregnancy-induced hypertension, or early-onset PE development [154]. 

Turi et al. noticed that women with pregnancy-induced HTN had arterial function parameters that were modified a long time before the first signs of high blood pressure (BP) occurred, and that BMI had a detrimental effect in all patients, especially in pregnant HTN women [155]. 

Mi et al. studied the second-trimester BP drop in predicting future pre-eclampsia. They observed that all subjects had a mid-pregnancy decrease in SBP, DBP, and MAP, and stated that BP trajectory throughout gestation could predict pre-eclampsia [156]. Moreover, insufficient uterine blood flow could lead to pre-eclampsia and foetal growth restriction [45]. 

Maternal pre-pregnancy obesity is connected to a range of complications during gestation, such as gestational diabetes, C-section, pre-term delivery, macrosomia and increased perinatal death [157]; the offspring of obese mothers also have consequences later in life. Sundholm et al. proved that the children of obese mothers had an enhanced body mass index and modified arterial function assessed through blood pressure and carotid intima-media thickness, and thus, an inherited CV risk factor [158]. 

The most significant predictors for elevated PWV and triglycerides/HDC-cholesterol ratio was proven to be maternal age, body mass index, gestational diabetes, systolic BP and indices of glucose metabolism [159]. 

Diabetic dyslipidaemia consisting of elevated triglycerides, decreased high-density lipoprotein (HDL) cholesterol, and a shift towards small dense low-density lipoprotein (LDL) is responsible for the increased CV risk in pregnant diabetic patients [160]. 

Enhanced HOMA-IR (Homeostatic Model Assessment for Insulin Resistance) appears to be the essential trigger for diabetic dyslipidaemia, and may modify the vascular function through the insulin-mediated proliferation of vascular smooth muscle cells and lipid synthesis, with subsequent LDL binding and extracellular matrix remodelling, and subsequent enhanced vascular stiffness [161]. Lekva et al. found that females with diabetes had a more increased risk for CVD 5 years after the index pregnancy; their status predicted the high TG/HDL-c ratio, the strong relationship between diabetes and systolic blood pressure predicted the PWV, and with BP and BMI, they could predict the Th/HDL-c ratio [159]. Sokup et al. recently reported that TG, HDL-C, and the TG/HDL-C ratio were elevated in diabetic females one year after the index pregnancy and may represent an early marker of endothelial dysfunction and CV risk [162], and that a combination of diabetes diagnosis with other risk factors such as BMI could identify people at enhanced CVD risk [163]. Levka et al. emphasised that women with gestational diabetes mellitus, defined according to the old WHO criteria, have an increased risk of future CVD, as estimated by increased arterial stiffness and more pronounced dyslipidaemia following pregnancy [159]. Retnakaran and Shah performed a large retrospective population-based cohort study of 435,696 women. They concluded that even in the absence of gestational diabetes mellitus, women with mild glucose intolerance in pregnancy (i.e., those with an abnormal 1 h glucose challenge test but a normal OGTT) have an elevated risk of CVD [164]. 

Although cardiac and metabolic disorders influence renal function and vice versa [165], and have shared epidemiological and pathophysiological features, this merged pathology called cardio-renal metabolic syndrome was studied as a whole only recently [166]. In a prospective study performed by Ishaku et al., it was concluded that hypertensive diseases in pregnancy are related to the increased incidence of metabolic syndrome up to one year after delivery, and that these women should be screened on a routine basis to check for metabolic syndrome and to reduce the cardiometabolic risk [167]. 

Arterial stiffness increases in pregnant women with diabetes compared to non-diabetic subjects, and their foetuses also have impaired CV parameters [168]. 

The PWV is crucial because it was demonstrated that in populations with high CV risk, aortic PWV is a valuable predictor for future outcomes, independent of the brachial arterial BP values [72]. Thus, although pregnant women can have acceptable BP values at regular check-ups, if their PWV is increased, it is a clear risk marker, and should be used as an essential tool for individualised monitoring and treatment plans [169]. 

The exact pathophysiological mechanisms are not known. Nonetheless, several theories have been considered. 

Coutinho conducted a review in which they studied the sex differences in arterial stiffness, as well as its potential underlying mechanisms and its role in the CVD in women [170]; they observed that women of more advanced age had increased aortic stiffness compared to men, thus leading to hypertension and insufficient control of the pathology, impaired diastolic function and ventriculoarterial coupling, left ventricle hypertrophy, CVD, and poor outcomes. Women tend to procreate at a more advanced age nowadays. 

Arterial wall compliance and stability depend on the balance between collagen and elastin, the extracellular matrix proteins. As we age, elastin decreases and collagen increases, thus leading to the stiffening of arteries. Moreover, if there are CV risk factors, inflammatory status and hormonal disbalance, this process becomes even more accelerated [171]. Hormones can have a protective role, such as oestrogen, which has been directly proven to affect arterial wall remodelling, increase elastin production, and decrease collagen in human arteries [172]. Pregnancy comes with modifications in the maternal body at all levels. Physiological pregnancies are defined by significant changes in the CV system, with enhanced LV mass, cardiac output and arterial compliance, and a decreased total vascular resistance. However, the data regarding myocardial and diastolic functions are conflicting [173]. Both arterial stiffness and peripheral vascular resistance decrease in the early gestation period, leading to enhanced plasma volume, so are necessary for healthy placental circulation and foetal development [174]. When the expected changes do not occur, this can lead to maladaptation, endothelial dysfunction, and increased arterial stiffness, with subsequent adverse outcomes for both the mother and foetus through the placental connection [168]. 

Particularly in pre-eclampsia, the most relevant mechanisms are the abnormal placenta and anti-angiogenic factors [35]. Altered placentation is the cornerstone of pre-eclampsia, and the findings in pathology exams included micro-thrombi, endothelial injuries, chronic inflammation and infarctions [175]. The standard process of placentation requires adhesion molecules to be expressed on the surfaces of the endothelial cells [176]. In pre-eclampsia, the uterine arteries do not follow the typical remodelling pattern; they become constricted and stiff, leading to hypoxia and the release of soluble factors, and thus generalised endothelial dysfunction [177]. Another essential role in arterial stiffness during pregnancy is played by vascular endothelial growth factors (VEGFs), responsible for vasculogenesis [178]. The placental growth factor is a VEGF homolog released by the placenta, with pro-angiogenic activity and the ability to potentiate the effects of VEGF, and a few studies emphasised that it might be a possible cause of endothelial dysfunction in pre-eclampsia [179]. 

In pre-eclampsia, the levels of sFlt-1 are increased, and those of PIGF are decreased even before the clinical signs occur [180,181], and in preterm pre-eclampsia, the levels of soluble endoglin (sEng)—another anti-angiogenic protein—enhance from 20 weeks of gestation [182]. The combination of sFlt-1, PlGF, and sEng levels foretells pre-eclampsia more accurately than any single marker, converging the combined actions of several angiogenic factors to produce the clinical phenotype of pre-eclampsia [182]. Furthermore, even if the sFlt-1 levels decline after the delivery of the placenta, a persistent and subtle anti-angiogenic milieu may be responsible for endothelial dysfunction and arterial stiffness in women with a history of pre-eclampsia. 

Nonetheless, although the consequences of enhanced maternal arterial stiffness on foetal–placental function have been demonstrated indirectly, the impact of the maternal arterial stiffens on foetal CV physiology has not been thoroughly studied. 

Unfortunately, to date, there are no randomised controlled multicentred and multi-ethnic studies on large cohorts evaluating the impact on morbidity and mortality in pregnant and postpartum women by integrating the measurement of arterial stiffness parameters into the basic assessments. Nonetheless, there are several studies that have shown the potential of integrating vascular function into pregnancy management in order to reduce morbi-mortality. Katsipi et al. proved that PWV could be used in predicting PE in high-risk women, and thus anticipating the morbidity and mortality in these women [169]. Turi et al. [29] concluded in a ten-year-long prospective study that there are significant differences between non-pregnant, healthy pregnant and pathological pregnancies, and arterial stiffness parameters (PWV, Aix). They concluded that arterial stiffness indices change before the first clinical signs appear, and thus, they emphasised that this could be used to prevent and manage these cases early, and that the BMI also had a deleterious effect in all three types of patients on the arterial stiffness. More recently, Anthoulakis et al. studied the correlation between PWV, Aix and pregnancies complicated by pre-eclampsia. They observed that PWV and Alx-75 are higher in pregnancies complicated by PE, compared to normotensive pregnancies, as well as in early-onset PE, compared to late-onset PE, and that arterial stiffness assessment represents a promising risk-stratification tool for future CV complications [183], but we are in need of future studies to confirm this. 

Other vital aspects regarding arterial stiffness and its implications in pregnant cohorts are related to the measurement methods and cut-off values.

In the beginning, arterial stiffness was measured through invasive methods. However, these measurements result from invasive pressure catheter recordings only used in technical validations, because, although precise and with a high temporal resolution, they are highly complex, expensive, inaccessible, invasive, and have ethical constraints [184]. Over time, more non-invasive methods have emerged, such as arterial tonometry, aortic ultrasound (transoesophageal echocardiography), carotid ultrasound and magnetic resonance imaging (MRI) [184]. MRI represents the only way to visualise the entire 3D aorta and its path length and times (phase-contrast sequences), and thus, offers a more appropriate evaluation of the PWV [185]. Carotid-femoral PWV (cfPWV) assessed by applanation tonometry and magnetic resonance imaging (MRI) are widespread techniques for PWV evaluation [186]. CfPWV is noninvasive, flexible, accessible, applicable to large populations, and inexpensive. However, the cons include the requirement of trained professional staff and its lack of preciseness, and it is not very accurate due to the inability to properly assess the whole aortic length, resulting in a systematic overestimation of PWV compared to MRI-PWV. Additionally, it cannot assess the local aortic pulse wave velocity, and obese patients can be extremely difficult to evaluate [74]. Brachial-ankle PWV, although non-invasive, automated, quick to assess and inexpensive, is ambiguous, and has limited correspondence to the aortic PWV. The same pros and cons apply for finger-toe PWV [184]. 

MRI faciliatates the precise, accurate non-invasive assessment of PWV; it is not widely available, requires local technical expertise, and is expensive and time-consuming, limiting its use in clinical care [74]. However, during pregnancy, both doctors and mothers avoid performing MRIs. Thus, the most utilised technique consists of arterial tonometry because it is accessible, easy to use, portable, safe, cheap, and the gold standard of aortic stiffness [147]. 

In order to appreciate the risk related to PWV, first, it is necessary to establish a cut-off value. Deciding the proper cut-off values for pregnant cohorts is one of the critical elements of this topic, because there is no consensus here. A cfPWV of 10 m/s is considered the proper cut-off for target organ damage in the general population [187]. Nonetheless, the risk appears to increase from a cfPWV of 7.8 m/s [146]. However, in women and [146] in pregnant female populations, there is no consensus regarding cut-off values. There are different versions, but not an established threshold, because to set it, it would be necessary to study the modifications in the female non-pregnant population thoroughly, and the pregnant population with and without pathology throughout the pregnancy and postpartum periods. 

Katsipi et al. proved that PWV yielded the highest detection rate of PE (81%) and early-onset (82%), and the sensitivity and specificity increased when sFlt-1 was added (90% and 92%, respectively), suggesting a cut-off value of 9 m/s for PWV resulted in optimal sensitivity (91%) and specificity (86%) for predicting early-onset PE [169]. However, we consider that more studies are necessary to establish a feasible threshold for pregnant women.

## 8. Discussion

In this review, we explored arterial stiffness in women with different medical backgrounds, especially pregnant women with risk factors or pathologies. 

Although quantifying arterial stiffness during gestation is of paramount importance in predicting hypertension development and other complications, there are several limitations to the existing studies. One of the most critical is that there is still no consensus regarding the cut-off values in the pregnancy category. It is not evaluated in all pregnant women [155], which is one crucial setback in all studies on this area. Furthermore, to date, there has been no comprehensive review of the available evidence on maternal arterial stiffness and its association with the outcomes during pregnancy. However, in 2022, Forrest et al. published a protocol for a systematic review and meta-analysis regarding the “arterial stiffness measurements in pregnancy as a predictive tool for hypertensive disorders of pregnancy and pre-eclampsia”, which hopefully will be an extensive research work [188]. As previously addressed, arterial stiffness and cardio-renal-metabolic modifications during pregnancy are interrelated and influence one another. However, there are still a lot of puzzles and incomplete data, such as PWV variability according to the measurement techniques, the gender disparities, and the constantly increasing maternal age. Females have a higher chance of having an unfavourable risk factors profile than men [189], and the cut-off values for PWV are also different. 

The limitations of the previous studies are varied. They range from the small size of the study group to the less precise PWV measurement methods, the lack of details regarding other possible physiological or pathological conditions that could influence the arterial stiffness, and the missing diversity in terms of region, social and economic background. Additionally, the lack of multicentred and multi-ethnic studies is a minus in this equation. There is also no focus on the entire family, the offspring, the partner, the detailed phenotyping, or genotype-wide genotyping. Moreover, most studies have focused on just one part of the pregnancy period: preconception, pregnancy, delivery, or postpartum. Assessing arterial stiffness only in one moment, for example, postpartum, without evaluating maternal hemodynamic performance status in the pre-pregnancy period can yield erroneous results, because vascular impairment before pregnancy cannot be excluded [190]. Only a few existing randomised controlled trials do not include all the data necessary to lead to guideline modifications. In 2021, Riemer et al. performed a prospective randomised controlled interventional trial starting six weeks postpartum regarding the effects of nutritional intervention combined with an intensive 6-month cardiovascular exercise programme on arterial stiffness through pulse wave velocity (PWV) in 38 women with severe hypertensive disorder of pregnancy (pre-eclampsia with or without pre-existing hypertension and/or HELLP syndrome) and compared it to a control group (postpartum women without pregnancy complications or CV risk) [191]. They measured PWV in both groups at delivery one week after in the control group and after six months in the study group. They observed that PWV in the study group corresponded to that of the reference group at the end of a study on lifestyle intervention with physical exercise after delivery (starting 6 weeks postpartum), emphasising that the intervention showed a significant clinical effect by reducing arterial stiffness to the level of the reference group [191]. However, this study was performed on a small cohort, and requires follow-up studies before it can confirm the results of this intervention in the middle term regarding CV risk. Before this type of intervention can be included in the standard of care and prevention, follow-up studies must confirm these results and the medium-term effects on cardiovascular risk. On this note, Jääskeläinen et al. published in 2022 a protocol for a future randomised controlled trial to assess the effectiveness of a 12-month lifestyle intervention to reduce cardiovascular disease risk in families ten years after pre-eclampsia (FINNCARE) [192]. There are other proposals for future RCTs [193], and we hope the results will lead to an adjustment of guidelines in this direction. 

Given the importance of CV prevention, extensive population-based studies are needed to confirm the independent additive role of PWV in order to identify which women might benefit from a regular follow-up. 

Another evolving area that is insufficiently explored is metabolomics, which is the next step that can be taken towards a different and more elaborated approach to arterial stiffness and its clinical implications. 

Carnitines are related to arterial stiffness in various situations. Studies conducted on patients with chronic kidney disease show that lower circulating levels of carnitine are associated with arterial stiffness. L-carnitine supplementation may increase N-oxide (TMAO), which predicts CVD through aortic stiffening, vascular inflammation, and altered cholesterol homeostasis [194]. Some more recent metabolomics studies associate arterial stiffness with enhanced acylcarnitine levels in various conditions, such as coronary artery disease (CAD) [195], insulin resistance [196], and type 2 diabetes [197]. Linoleic acid was found to be a significant independent determinant of arterial stiffness. Lower serum docosahexaenoic and eicosapentaenoic were associated with increased arterial stiffness [198]; higher arachidonic, eicosapentaenoic and docosahexaenoic, and lower oleic, palmitic, and linoleic acid levels were linked to decreased PWV and systolic BP [199]. Lower serum levels of phosphatidylcholines were related to increased arterial stiffness, increased resting heart rate, or endothelial dysfunction [200], and the pro- or anti-atherogenicity of lysophosphatidylcholine probably depends on the physical properties of its fatty acid residue. In a large-scale study of female twins, the sphingolipid precursor Ser was associated with PWV [201]. Ceramides, glycosphingolipids, and S1P were proven to be involved in atherogenesis, and ceramides were proven essential in increasing PWV [202]. Circulating lactosylceramide was proven an independent predictor of enhanced arterial stiffness in subjects with impaired fasting glucose [203]. A module composed of monoacylglycerol and diacylglycerol, among other glycerolipids, was associated with both AIx and PWV [204]. Higher circulating levels of indoxyl sulfate, a uremic toxin that is produced by the metabolism of dietary tryptophan, showed positive associations with aortic stiffness in patients with CAD [205] and type 2 diabetes [206]. Higher homocysteine levels are associated with arterial stiffness via direct effects (smooth muscle proliferation, ED, collagen synthesis, and elastolysis) on the arterial wall [207,208]. It appears that interventions such as diet and exercise could influence arterial stiffness from a metabolomic perspective [209]. Besides this, medical management could be more effective if it included metabolomics-targeted treatment strategies (and the treatment would be more individualised). Measuring circulating acylcarnitine, glycerophospholipids, sphingolipids and amino acids, known as arterio-metabolites, and assessing the arterial stiffness through non-invasive methods such as PWV, can lead to a more accurate risk calculation, individualised treatment plan and disease monitoring [210]. Emerging research proves the importance of arterio-metabolites in refining the risk and elaborating the treatment plan. 

For pre-eclampsia, adding the sFlt-1/PlGF ratio can significantly increase the sensitivity and specificity of the PWV in predicting pre-eclampsia. 

Another critical aspect that is becoming obvious is related to the increasing maternal age, and the fact that the cut-off value of 35 y will probably be modified towards higher ages. In the latest INSEE report from France, it was stated that approximately 5% of women who give birth are at least 40 y old, hence the idea of pushing the limit for advanced maternal age to 43–45 y old [211]. 

The economic burden of pre-eclampsia on health care systems is estimated at 40–100 times the costs of term pregnancy, depending on gestational age at delivery [212]. The implementation of a first-trimester screening program for pre-eclampsia and early intervention with aspirin in women identified as high-risk for early PE has the potential to prevent a great number of early-onset PE cases, with a substantial associated saving of costs to the health care system [213]. 

The risk factors that lead to pre-eclampsia, such as obesity, unhealthy diet, lack of exercise, high blood pressure, deglycation, dyslipidaemia and abnormal renal function, all contribute to the dysbalanced cardio-renal-metabolic chain, and in this vicious circle, each component enhances the other with catastrophic outcomes [35]. 

Nonetheless, these risk factors and pathologies have a common denominator: arterial stiffness, which has a role in predicting their occurrence and in the outcomes, and is an easy, inexpensive, reliable, painless, non-invasive, non-radiant and reproducible method [214]. Arterial stiffness measurement should be a mandatory mode of dynamic assessment, in the form of a continuous follow-up starting from before (pre-pregnancy), and continuing during (each pregnancy trimester) and after, a gestation (at least 6 weeks postpartum and after 1 year and longer) to quickly detect and prevent the mother and offspring from suffering lifelong consequences, because pregnancy should be regarded as a global public health priority.

## 9. Conclusions and Future Perspectives

Arterial stiffness measured through PWV predicts the outcomes in pregnant women for both mother and foetus in the short and long run. Nevertheless, more questions remain to be answered: what is the appropriate cut-off value for pregnant women regarding the PWV? What is the most accurate and safest technique that can be employed in pregnancy to assess arterial stiffness? What additional data can stratify the risk in these women? When and how often should they be evaluated from this point of view? What are the modalities to stop or reverse arterial stiffness and its implications? What are the implications of increased arterial stiffness for the mother and foetus in the short and long term? 

In order to answer these questions, we need well-designed, multi-centred, multi-ethnic, large randomised controlled trials, which should include patients from all social backgrounds from the earliest stages, meaning from the pre-gestational age, and having their arterial function carefully evaluated. Then, we should assess them in the gestational period for at least four periods: first, second and third trimester, and postpartum (at least 6 weeks). Afterwards, we should continue with the follow-ups for years in both mother and offspring. Moreover, besides the classical laboratory assessment (total cholesterol, low-density lipoprotein cholesterol, high-density lipoprotein cholesterol, triglycerides and glucose), newer biomarkers called arterio-metabolites (low-molecular-weight metabolite (LMWM) species—L-Carnitine, Trimethylamine N-Oxide, and Acylcarnitines) added to arterial stiffness measurements (PWV and Aix) could help us further interrogate the human metabolome and vascular system. The data provided in the previous chapters open the door to the complex cardio-renal–metabolic chain and the implications of arterial stiffness and pregnancy in this circle. Adding the increasingly growing data regarding the impact of arterial stiffness on overall health, including during pregnancy, the constantly evolving understanding of arterial stiffness and metabolomics could lead to the occurrence and validation of novel biomarker panels with a predictive capacity more significant than the conventional CV markers. Therefore, in everyday clinical practice, a more detailed overview of subclinical metabolic and hemodynamic (e.g., increased cf-PWV) alterations could be helpful for patients in whom traditional biomarkers/parameters give borderline or equivocal results. It could also help us monitor disease (e.g., obesity, chronic kidney disease, hypertension, diabetes) progression and improve management efficacy.

Moreover, adding metabolomics to arterial stiffness measurements could emerge as a novel therapeutic target for vascular ageing. 

Another helpful research approach should consist of conducting multiple systematic reviews and meta-analyses. 

Because PWV could be ameliorated through lifestyle changes and/or medical treatment of the RFs, it has the potential to be not only a diagnosis and prognosis marker, but also an objective for the proper management of a surrogate endpoint in genomic studies and clinical trials, for a better understanding of the relarionsip between the genotypes and phenotypes in the CV renal–metabolic chain and personalised medicine in pregnant patients. 

The literature is scarce on research regarding the impact of arterial stiffness in pregnant cohorts and the implications for the cardio-renal metabolic syndrome, and that which does exist focuses on small groups. Thus, further studies are necessary to implement this into routine use in clinical practice, because arterial stiffness assessment is a promising risk-stratification tool for future cardiovascular complications.

## Figures and Tables

**Table 1:** Classification of weight status according to BMI [100,101].

BMI (kg/mp^2^)	Classification
≤18.5	Underweight
18.5–24.9	Normal/healthy/average
25.0–29.9	Overweight
30–34.9	Obese I
35–39.9	Obese II
≥40	Obese III

**Table 2:** Risk factors for pre-eclampsia (PE).

Minor Risk Factors	Major Risk Factors
Advanced maternal age	Pre-existing chronic hypertension
Nulliparity	Renal disease
Family history of PE	Personal history of PE
Short and long inter-pregnancy intervals	Autoimmune disease
Assisted reproductive technologies	
Obesity	
Ethnicity	
